# Maternal sensitivity to distress, attachment and the development of callous‐unemotional traits in young children

**DOI:** 10.1111/jcpp.12867

**Published:** 2018-01-30

**Authors:** Nicola Wright, Jonathan Hill, Helen Sharp, Andrew Pickles

**Affiliations:** ^1^ Institute of Psychology, Health and Society University of Liverpool Liverpool UK; ^2^ School of Psychology and Clinical Language Sciences University of Reading Reading UK; ^3^ Biostatistics Department at the Institute of Psychiatry King's College London London UK

**Keywords:** callous‐unemotional (CU) traits, parenting, infancy, attachment

## Abstract

**Background:**

Callous‐unemotional (CU) traits are characterized by a lack of responsiveness to the emotions of others, particularly negative emotions. A parenting environment where the child's own distress emotions are sensitively responded to may help foster the child's ability to respond to the emotions of others. We tested whether maternal sensitivity to distress, and other parenting characteristics, were associated with CU traits over the preschool period, and examined whether this was mediated via infant attachment status.

**Method:**

In an epidemiological cohort, CU traits were assessed at age 2.5, 3.5, and 5.0 years by mother report. Dimensions of parenting were assessed in free play at age 29 weeks in a stratified subsample of 272, and attachment status at 14 months (*n* = 265). Structural equation modelling with maximum likelihood estimation was used to examine predictions from parenting dimensions and attachment status.

**Results:**

A parenting factor comprised of sensitivity to distress (*n* = 207), sensitivity to non‐distress, positive regard toward the infant (or warmth), and intrusiveness, predicted child CU traits (*p *=* *.023). This effect was accounted for mainly by sensitivity to distress (*p *=* *.008) and positive regard (*p *=* *.023) which showed a synergistic effect as evidenced by a significant interaction (*p *=* *.01). This arose because the combination of low sensitivity to distress and low positive regard created the risk for elevated CU traits. Although sensitivity and positive regard predicted attachment security and disorganization, there were no associations between attachment status and CU traits.

**Conclusions:**

The finding of contributions from both sensitivity to distress and positive regard to reduced CU traits suggests that children's responsiveness to others’ emotions may be increased by their own mothers’ responsiveness to them and their mothers’ warmth. There was no evidence that this was mediated via attachment status. Implications for intervention and future directions are discussed.

## Introduction

There is much current interest in a possible subgroup of conduct disordered children who show a lack of concern for the feelings of others and lack of guilt or remorse, labelled as ‘callous‐unemotional traits’ (CU traits) (Frick, 2009). There is some evidence that there may be distinct developmental processes contributing to the development of conduct problems with and without CU traits. Conduct problems in children with CU traits have been found to be more highly heritable (Viding, Jones, Frick, Moffitt, & Plomin, 2008), less influenced by negative parenting practices (Pasalich, Dadds, Hawes, & Brennan, 2012), and less responsive to typical conduct problem interventions (Hawes, Price, & Dadds, 2014). CU traits have been linked to more severe and stable antisocial behavior in childhood (Frick, Ray, Thornton, & Kahn, 2014) and of particular interest is the association with physical aggression, with CU traits being associated with more severe violent and aggressive behavior (Kruh, Frick, & Clements, 2005).

Evidence from several prospective general population based studies of children aged 2 years and older points to the possibility that aspects of positive parenting contributes to lower CU traits. These have included studies of self‐reported positive reinforcement and parental involvement (Hawes, Dadds, Frost, & Hasking, 2011), parental warmth assessed using the five minute speech sample (FMSS) and observations of parenting in the home (Waller et al., 2014). Using an index of parental sensitivity derived from parent–child observations at ages 24, 36, and 58 months, Wagner, Mills‐Koonce, Willoughby, Zvara, and Cox (2015) found that less sensitive parenting predicted higher levels of CU traits in first grade controlling for earlier measures of CU behaviors. We have previously reported that maternal sensitivity assessed at age 29 weeks predicted CU traits at 2.5 years (Bedford, Pickles, Sharp, Wright, & Hill, 2015), and Centifanti, Meins, and Fernyhough (2016) found that mind‐mindedness, indexing the mother's awareness of her infant's states of mind, assessed at age 8 months predicted children's self‐report of CU traits at 10 years.

As Mesman and Emmen (2013) showed in their meta‐analysis, there has been considerable variability in the ways parental sensitivity has been conceptualized and measured. Mary Ainsworth's original coding system focused on the extent of well‐timed maternal responses to infant cues, and did not assess maternal warmth, however, subsequent measures have commonly included both in the sensitivity construct (e.g. Feldman, 1998). Similarly, sensitivity to infant distress and to infant cues while not distressed, may support different infant capabilities and predict different outcomes (Leerkes, 2011; McElwain & Booth‐LaForce, 2006; Murray et al., 2008). Thus, although scores on the dimensions of sensitivity to distress and to non‐distress, and of warmth/positive regard, are correlated, assessing their distinctive contributions may be informative in relation to early mechanisms for CU traits. Sensitivity to distress may specifically promote empathy which is a core construct for CU traits (Jones, Happe, Gilbert, Burnett, & Viding, 2010), via processes such as modelling (Kiang, Moreno, & Robinson, 2004) or imitation (Baird, Scheffer, & Wilson, 2011). Davidov and Grusec (2006) have previously argued for a specific link between responsiveness to distress and child empathy. In a cross‐sectional study of 6–8 year olds, higher maternal sensitivity to distress, but not warmth, was associated with increased child empathy. In a randomized controlled trial of the effect of foster care in children experiencing early institutional deprivation, observed sensitivity to distress, but not warmth, assessed at 30 and 42 months of age, predicted lower CU traits in early adolescence (Humphreys et al., 2015).

The contingent responding to infant gestures characteristic of high sensitivity may contribute specifically to increasing eye contact between infant and parent. This may mitigate the reduced eye contact found in children with CU traits and hence enhance empathic responding (Dadds et al., 2006, 2014). The finding that a reduced preference for the human face compared to inanimate objects over the human face at 5 weeks of age is associated with CU traits at age 2.5 years (Bedford et al., 2015) suggests this may operate early in development (Bedford et al., 2017).

Sensitivity to distress may also be important by virtue of its association with attachment status. A possible role for attachment processes was indicated by the finding in Wagner et al. (2015) that the association between low parental sensitivity and CU traits was mediated in part by scores for dysfunctional family representations derived from children's drawings of their families completed in first grade. Thus empathy, and hence lower CU traits, may be promoted by internalization of the experience of empathic responding by parents. Evidence for the role of attachment status in relation to CU traits comes from a study of 3–9 year olds referred with conduct problems (Pasalich et al., 2012). Higher CU traits were associated with insecure and with disorganized attachment, based on the Manchester Child Attachment Story Task, a story completion task in which children are asked to portray resolutions of attachment challenges such as being frightened in the night (Green, Stanley, Smith, & Goldwyn, 2000). Willoughby, Mills‐Koonce, Gottfredson, and Wagner (2014) showed that attachment disorganization assessed at 3 years was associated with a stronger association between the combination of ODD and CU traits and aggression, but did not examine its association with CU traits in multivariate analysis.

Overall the available evidence suggests that aspects of positive parenting in early childhood are associated with lower CU traits, however, little is known about the role of parenting during infancy, and the contributions of specific dimensions of parenting have not previously been examined. Furthermore, the question of whether infant attachment status mediates any associations has not been previously addressed. In this study, we examined specificity of parenting dimensions by comparing contributions from a general parenting factor as well as direct pathways from each separate parenting dimension in Structural Equation Modelling (SEM). SEM also allowed us to generate a robust indicator of CU traits as the outcome derived from measurement at 2.5, 3.5, and 5.0 years. On the basis of available evidence, we predicted that maternal sensitivity, and not an index of warmth (i.e. positive regard), would be associated with lower CU traits. In view of several lines of evidence that sensitive responding to distress may promote empathy we predicted that the effect of maternal sensitivity would be specific to mothers’ responses to distress. We also examined whether the associations between maternal sensitivity and CU traits were mediated via infant attachment status.

## Methods

### Sample

Participants were members of the Wirral Child Health and Development Study, a prospective epidemiological cohort study starting in pregnancy. The cohort consists of 1233 first‐time mothers who had live singleton births. Socioeconomic conditions on the Wirral range between the deprived inner city and affluent suburbs, but with very low numbers from ethnic minorities. Mean age of the mothers at recruitment was 27.9 years (*SD *= 6.2, range 18–51), 42% of the sample were in the most deprived quintile of UK neighborhoods (Noble et al., 2004) and 96% were White British.

The measures used in this report were obtained for the whole cohort from questionnaires at initial recruitment at 20 weeks gestation and ratings of the child behavior when aged 3.5 years (*M *=* *41.89 months, *SD *= 2.5; *n* = 827) and 5.0 years (*M *=* *58.64 months, *SD *= 3.7; *n* = 775). Additional measures were obtained for a random subsample stratified by psycho‐social risk of mothers (*n* = 316) who were to provide interviews at 32 weeks gestation (*M *=* *32.1, *SD *= 2.0) and mother–infant observational measures with the child aged 29 weeks (*M *=* *29.1 week, *SD *= 3.1; *n* = 272) and 14 months (*M *=* *14.3 months, *SD *= 1.9; *n* =  268) and additional ratings of the child behavior when aged 2.5 years (*M *=* *31.11 months, *SD *= 2.67; *n* = 253). The stratified sampling has been described in more detail previously (Sharp et al., 2012) and analyses included the stratification variable, psychological abuse in the partner relationship (Moffitt et al., 1997), to adjust for effects associated with the relative oversampling of mothers with high psycho‐social risk.

The sample analyzed here comprises all participants who provided observational data at age 29 months (*n* = 272). This subsample was a relatively even mix of boys (*n* = 134) and girls (*n* = 138). At age 5.0, 80% of mothers were either married or cohabiting, 5% had a partner living elsewhere and 15% were single.

### Ethical considerations

All women gave written informed consent at the point of recruitment in the antenatal clinic. Ethical approval for the study was granted by the Cheshire North and West Research Ethics Committee on the 27 June 2006.

### Measures

#### Maternal sensitivity

Mother–child interactions at 29 weeks were videotaped during a semi structured 15‐min play session in a purpose built room in the study base. Mother–infant dyads played with a toy of the mother's choice for the first 7 min and with a standard set of toys provided by the experimenter for the following 8 min (as described in National Institute of Child Health and Human Development – Early Childcare and Youth Development NICHD‐ECCRN, 1999). The interactions were coded for maternal sensitivity to nondistress and to distress, positive regard, and intrusiveness using the NICHD manual (Owen, 1992). All the parenting codes are rated on a global 5‐point scale, ranging from 1 (*not at all characteristic*) to 5 (*highly characteristic*). Sensitivity to distress captured the extent to which the mother responded to her infant's cries, frets or distress in a consistent, timely, and appropriate manner. Sensitivity to non‐distress captured the extent to which the mother observed and responded in a well‐paced and appropriate manner to her infant's social gestures, expressions, and signals of non‐distress. Positive regard captured the parent's positive feelings toward the child expressed during the interaction, shown by behaviors such as smiling at the child or laughing with the child. Intrusiveness captured the extent to which the interaction is adult centered rather than child centered, shown by behaviors such as not allowing the child to handle toys that they reach for or insisting that the child do something (play, eat, interact) in which they are not interested.

In addition to coding sensitivity to distress on the 207/272 children who showed distress, we also rated the duration of the distress on which this was based (inter‐rater reliability based on 20 recordings, ICC = .92). This information has not been provided in previous studies using this scale but may be important for comparison across studies, and because validity may be compromised at lower durations of distress. In view of the evidence from this and other studies that sensitivity to distress reflects a general sensitivity construct, as well as maternal responsiveness specifically to distress, we used correlations between sensitivity to distress with sensitivity to nondistress as an index of construct validity. To provide an estimate of whether validity may be lower with shorter durations of distress, we calculated the correlations at each of the quartiles of duration of infant distress. Training on the sensitivity measure was provided by an investigator from the NICHD Network. Three raters, blind to the other measures, coded sensitivity from video recordings. Each rater achieved good inter‐rater reliability for maternal sensitivity, positive regard and intrusiveness on a subset of 30 assessments (ICCs .83–.89). Ratings were log transformed to minimize skew and standardized to aid effect comparison.

#### Attachment security

Infant–mother attachment was assessed at 14 months using the Strange Situation Paradigm (Ainsworth, Blehar, Waters, & Wall, 1978). The Strange Situation is a widely used laboratory procedure designed to assess the attachment relationship between infants aged 12–20 months and a caregiver. One trained rater who was blind to all other study data coded all infant–mother strange situations, and assigned them as Secure, Avoidant, Resistant or Disorganized. To evaluate inter‐rater reliability, 53 strange situations (20%) were selected randomly for coding by a second trained rater who was also blind to the study details. The two coders achieved inter‐rater reliability on the four‐way classification (81% exact agreement; kappa = .72) coding schemes. A total of 268 children in total completed the strange situation paradigm, of which three were assigned ‘cannot classify’ and were not included in analyses. In the four‐way classification, 128 (48%) of children were secure, 87 (33%) were disorganized, 27 (10%) were avoidant and 23 (9%) were resistant. For this analysis, we created two binary variables: secure = 0/insecure = 1 and organized = 0/disorganized = 1.

#### CU traits

CU traits were assessed by mother‐report at 2.5, 2.5, and 5.0 years using a combination of the Antisocial Processes Screening Device (Frick & Hare, 2001) and items from the Child Behavior Checklist (CBCL; Achenbach & Rescorla, 2000), the Brief Infant Toddler Assessment (BITSEA; Briggs‐Gowan, Carter, Irwin, Wachtel, & Cicchetti, 2004) and the Strengths and Difficulties Questionnaire (SDQ; Goodman, 1997). We have previously created CU traits latent factor scores at age 2.5 and 5.0 years (Wright, Sharp, Pickles, & Hill, 2016) by subjecting items to exploratory and confirmatory factor analyses in MPlus (Muthén & Muthén, 2012). For this study, we applied the same process to the age 3.5 year items (see Appendix S1). We allowed the items at each age to vary to reflect developmental differences in the manifestation of CU traits, the items for each age are displayed in Table S1. The age 3.5 CU traits measure showed factorial invariance by sex (see Table S2) and the CU items were distinct from physical aggression items (see Table S3). For this analysis, a latent variable was created from the three factor scores to represent CU traits from age 2.5 to 5.0 years. The summary statistics and the associations between the three age points are displayed in Tables S4 and S5.

#### Covariates

Covariates reflected family demographic status, partner psychological abuse at entry to the study to account for the stratification, maternal mood at times of reporting of CU traits to account for possible mood based reporting biases, and infant fear because of evidence that elevated fear may be a risk for later CU traits (e.g. Waller et al., 2016). Two indices of family demographic status were included as covariates: (a) socioeconomic status, which was derived from post code data using the English Index of Multiple Deprivation (IMD; Noble et al., 2004) and converted to quintile categories with a binary variable (1 = most deprived, 0 = all 4 other quintiles) used for analysis and (b) mother's age at consent. The stratum variables indicating stratification status created from the partner psychological abuse measure (Moffitt et al., 1997) were included as covariates. Mother's depression at time of reporting CU traits was assessed at age 2.5 and 3.5 years using the Edinburgh Postnatal Depression (EPDS; Cox, Holden, & Sagovsky, 1987) and at 5.0 years with the Centre for Epidemiological Studies Depression Scale (CES‐D; Radloff, 1977). A standard score was created at each age and a mean score of the three time points was used for analysis. Infant fear at age 29 weeks was assessed using the unpredictable mechanical toy task from the Laboratory Temperament Assessment Battery (Lab‐TAB; Gagne, Van Hulle, Aksan, Essex, & Goldsmith, 2011). In this task, the infant is exposed to an unpredictable mechanical toy for 60 s, each 10 s epoch is coded on a 3‐point scale for facial, bodily and vocal fear, and escape behaviors, and a mean score across all epochs is used for analysis. Two raters, blind to the other measures, coded the Lab‐TAB from video recordings. Acceptable reliability was achieved on a subset of 30 assessments (ICC = .74).

### Analysis plan

All analyses were conducted in Stata version 14 (Statacorp, 2015). The main analyses used SEM using the *sem* and *gsem* commands, the latter being required for models that included the binary attachment status outcomes, with maximum likelihood estimation. The analyses proceeded by first examining prediction from each NICHD parenting code (sensitivity to distress, sensitivity to nondistress, positive regard, and intrusiveness) to attachment status and to child CU traits. Then, the four parenting variables were modelled as a general parenting latent variable and prediction using this general parenting factor was examined. If prediction from the factor was shown, further SEM models were then estimated with a direct path added from each parenting variable to test for specificity of prediction among the four parenting measures. We then examined the prediction of CU from attachment and the four parenting measures, for the latter following the same procedure as for the prediction of attachment.

CU traits were modelled as a latent variable based on measurement at ages 2.5, 3.5, and 5.0 years to increase statistical power, and to avoid multiple analyses for each outcome point. To check whether the association between maternal caregiving and CU traits might have been weaker for the latest measure of CU traits at age 5 years than for the latent variable, we tested the significance of additional direct paths to the most distal CU measurement at age 5.0.

Since we wished to make inference about all mothers and infants, and not just those with distressed infants, we needed to include in the analysis all dyads, regardless of distress status. Maximum likelihood modelling of the general parenting factor also allowed us to tackle this problem of an absence of a measure of sensitivity to distress whenever the infant failed to show distress during the observation, under an assumption of missing‐at‐random. This allowed the probability of such missingness to be associated with a parent's sensitivity to nondistress, positive regard, and intrusiveness, as well as included covariates and stratifiers. To examine each individual contribution of each parenting indicator in turn, the error variance of each indicator was in turn set to zero so that the factor reflected each specific indicator one at a time.

We examined for possible synergy among parenting indicators identified as important in the prediction of child CU traits. We calculated the product of centered scores from two parenting indicators as an additional indicator of the factor and, as above, examined whether there were additional effects from this product indicator along a direct path to the CU traits factor. The same approach was also taken to examine whether attachment insecurity or disorganization moderated the association between parenting variables identified as important and later child CU traits.

Model fit using the *sem* command was assessed using the Root Mean Square Error of Approximation (RMSEA) and the Comparative Fit Index (CFI). RMSEA <.05 and CFI >.95 are indicative of good fit whereas RMSEA <.08 and CFI >.90 represent reasonable fit (Hau, Marsh, & Wen, 2004). Stata does not produce fit statistics for *gsem* models, so for these models we relied on the size and significance of the estimates alone.

## Results

The simple correlations and summary statistics for all the variables are presented in Table 1. It can be seen that maternal sensitivity to distress, sensitivity to nondistress, and positive regard were strongly correlated, with intrusiveness showing weaker but still substantial correlations with the other parenting variables. Lower maternal sensitivity, lower positive regard, and higher intrusiveness, were associated with being younger at the time of first child, being exposed to partner psychological abuse during pregnancy (sample risk stratifier), and living in an area of high deprivation, underlining the importance of controlling for these variables in all subsequent analyses.

**Table 1:** Summary statistics and bivariate associations (Spearman's rho) between main study variables and covariates

	CU factor	Distress	Nondistress	Intrusive	Pos. Regard	Insecure	Disorg.	Infant fear	Mat. Dep.	Risk	Mat. age	Deprived
Sensitivity distress	−.27***											
Sensitivity nondistress	−.19**	.72***										
Intrusiveness	.09	−.38***	−.50***									
Positive regard	−.25***	.71***	.81***	−.32***								
Insecure attachment	.07	−.11	−.11	.05	−.11****							
Disorganized attachment	−.01	−.12	−.12****	.09	−.14*	.31***						
Infant fearfulness	−.04	.03	−.01	.02	.01	−.04	.09					
Mothers depression	.17**	−.01	−.06	.02	−.10	.05	.04	.03				
Sample risk stratum	.16**	−.15*	−.16*	.13*	−.12****	.05	.13*	.01	.18*			
Maternal age	−.19***	.31***	.39***	−.20**	.33***	.01	−.03	−.07	−.06	−.17**		
Deprived	.09	−.20***	−.24***	.16**	−.23***	.02	.09	−.01	.02	.08	−.31***	
*N*	272	207	272	272	272	265	265	272	271	272	272	272
Mean (*SD*)	−0.01 (0.29)	3.42 (1.00)	3.70 (0.99)	1.89 (0.87)	3.60 (0.91)	0.62 (0.49)	0.33 (0.47)	0.41 (0.33)	−0.01 (0.84)	0.76 (0.78)	27.78 (6.18)	0.38 (0.49)

***p <* *.001; **p <* *.01; *p <* *.05; ****p <* *.10.

### Durations of infant distress in the NICHD play assessment

Among the 207 infants who showed distress, the average duration of distress was 129.86 (*SD *= 115.90) seconds which represented an average of 14.7% (*SD *= 13.6%) of the 15 min assessment period. The association between sensitivity to distress and sensitivity to nondistress used as an index of construct validity was *rho *= .71, *p *<* *.001.^1^ Associations in each quartile of the distribution of duration of distress as percentage of the assessment period were: Lowest quartile (range .22% to 3.67%) *rho *= .70 *p *<* *.001, 2nd quartile (range 3.68% to 10.78%) *rho *= .75 *p *<* *.001, 3rd quartile (range 10.79% to 22.56%), *rho *= .64, *p *<* *.001, highest quartile (range 22.57% to 67.08), *rho *= .67, *p *<* *.001. Thus there was no evidence that the strengths of association between sensitivity to distress and to non‐distress varied by duration of distress, supporting the validity of the sensitivity to distress measure even at shorter durations.

### Parenting to attachment status

Models predicting binary attachment status used the *gsem* command and produced unstandardized probit coefficients. Examining the effects of each indicator in turn showed that sensitivity to distress was associated with insecure attachment (est = −0.18, *p *=* *.046, 95% CI [0.01, 0.36]), and there were similar but marginal effects for positive regard (*p *=* *.068) and to a lesser extent sensitivity to nondistress (*p *=* *.104) and nonsignificant effects in the opposite direction for intrusiveness (*p *=* *.424). The factor formed by the four parenting indicators together (with a negative factor loading for intrusiveness), while giving a reasonable model fit (RMSEA = .04, CFI = .99), showed only a marginally significant effect on insecure attachment (*p *=* *.079).

Corresponding analyses for disorganized attachment gave an identical pattern of findings, with low sensitivity to distress a significant predictor (est = 0.21, *p *=* *.024, 95% CI [0.02, 0.40]), and similar effects of low positive regard (*p *=* *.061), low sensitivity to nondistress (*p *=* *.173) and intrusiveness (*p *=* *.362). Here again, the parenting factor's effect was similar to that of the individual measures and of marginal significance. (*p *=* *.083).

### Prediction of CU traits from attachment and parenting

We fitted a confirmatory factor analysis model to the CU traits measurements at age 2.5, 3.5, and 5.0 year (see Appendix S1 for a description of their construction). The model showed good fit (RMSEA = .00, CFI = 1.00) with factor loadings of .69, .80, and .67 for age 2.5, 3.5, and 5.0 years, respectively.

**Figure 1:**
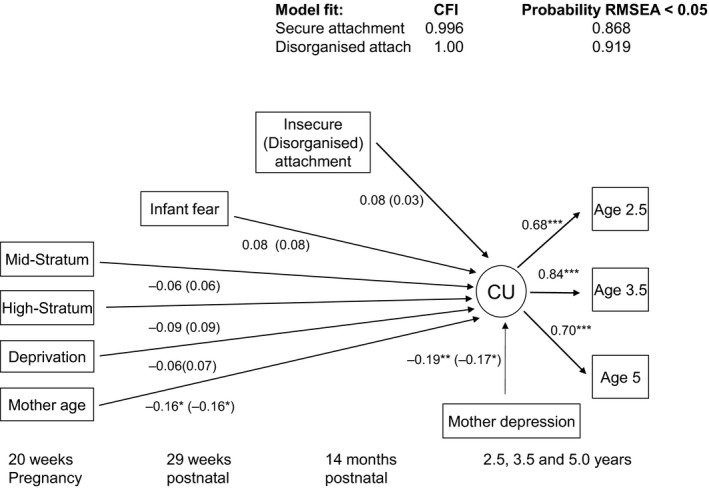
Standardized estimates for insecure attachment model and disorganized attachment model (in parentheses) predicting child CU traits. Note. *p < .05; **p < .01; ***p < .001

**Figure 2:**
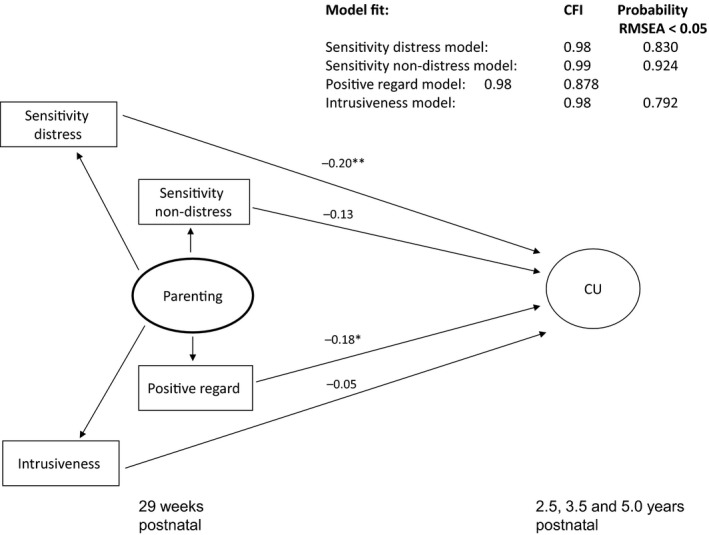
Standardized estimates for each parenting indicator predicting child CU traits. Note. *p < .05; **p < .01; ***p < .001. This figure depicts the results of four separate *sem* models

Models for the prediction of CU traits by attachment status fit well but, as shown in Figure 1, neither insecure nor disorganized attachment made independent contributions (secure: *p *=* *.265; organized: *p *=* *.652). Figure 2 shows the results from the models considering each of the parenting indicators as predictors of CU traits in turn. Sensitivity to distress (*β *= −.20, *p *=* *.008, 95% CI [−.34, −.05]) and positive regard (*β *= −.18, *p *=* *.023, 95% CI [−.33, −.03]) were associated with lower CU traits, with both models explaining 14% of the variation in the CU traits factor. There was a similar but nonsignificant effect of sensitivity to nondistress (*β *= −.13, *p *=* *.088, 95% CI [−.27, .02]). The effect of intrusiveness was much smaller and nonsignificant (*β *= −.05, *p *=* *.461, 95% CI [−.19, .08]). These models explained 12% and 11%, respectively, of the variation in the CU traits factor.

**Figure 3:**
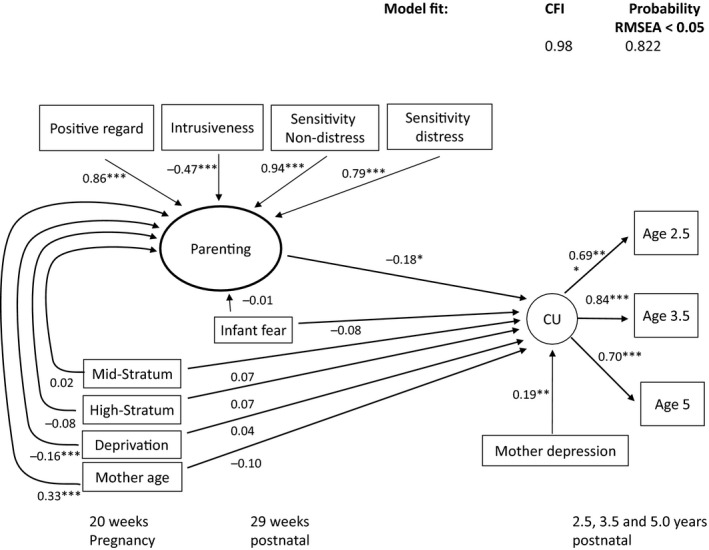
Standardized estimates for the latent parenting factor predicting child CU traits. Note. *p < .05; **p < .01; ***p < .001

The general positive parenting factor formed by the four indicators together significantly predicted lower CU traits (*β *= −.18, *p *=* *.023, 95% CI −.33, −.03) explaining 13% of the variation in the CU factor. This model, shown in Figure 3, was then extended in two ways to clarify the prediction of CU traits. The first examined whether any aspects of parenting showed a particular association with CU traits beyond that implied by their contribution to the general parenting factor, by testing for the effect of including the specific pathway from each parenting variable on the CU traits factor. The addition of either the sensitivity to distress or positive regard direct pathways rendered the effect of the parenting factor nonsignificant, suggesting that each contributed substantially to the effect of the factor. When added to the effect via the parenting factor, the direct pathway was significant for positive regard (*p *=* *.036), but not sensitivity to distress (*p *=* *.165). The addition of the intrusiveness and sensitivity to nondistress pathways had little impact on the prediction from the parenting factor to CU traits, indicating that they did not make major contributions to its effect on CU traits, though the estimates for the latter model showed collinearity problems.

To examine the stability of the association of early parenting with later CU traits, specifically addressing the question that it might decline over time, we tested for differential association (additional direct path) to the most distal CU traits measurement at age 5.0. Neither that from the parenting factor as a whole (*p *=* *.195), nor those from any of the four components (sensitivity to distress: *p *=* *.697, positive regard: *p *=* *.164, sensitivity to nondistress *p *=* *.183 and intrusiveness: *p *=* *.462) were significant. There was thus no evidence to suggest that the effects found dissipated over time.

**Figure 4:**
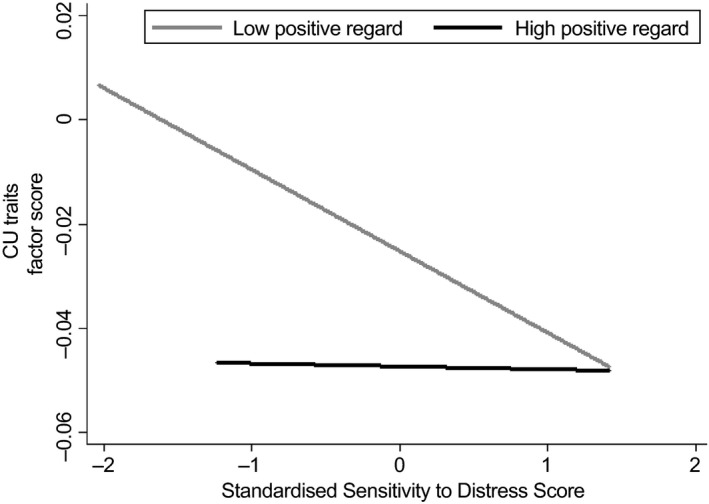
Plot showing prediction of CU traits from sensitivity to distress at high and low positive regard groups, divided at the mean score

As analyses of the parenting indicators separately, and in relation to the parenting factor, had indicated roles for sensitivity to distress and positive regard, we examined whether they had a synergistic effect by including an additional indicator formed by the interaction between sensitivity to distress and positive regard. The additional path from the interaction term to the CU factor was significant (*p *=* *.010; Model fit: RMSEA = .05, CFI = .96), and raised the explained variance of the CU factor to 17%. The effect of the interaction is shown in Figure 4 contrasting effects of sensitivity to distress in groups below and above mean positive regard. It can be seen that high CU traits were predicted by the combination of low positive regard and low sensitivity to distress, but not by either one of these in the absence of the other. A final check showed that this interaction had no role in the prediction of insecure or disorganized attachment.

Finally, we examined whether attachment status moderated the association between maternal caregiving and child CU traits by adding an interaction term to the models testing prediction from the overall parenting factor and the two key parenting variables, sensitivity to distress and positive regard. None of the models showed a significant interaction (parenting factor X disorganized: *p *=* *.211; parenting factor X secure: *p *=* *.818; sensitivity to distress X disorganized: *p *=* *.318, sensitivity to distress X secure: *p *=* *.553, positive regard X disorganized: *p *=* *.727, positive regard X secure: *p *=* *.144) providing no evidence for moderation by attachment status.

## Discussion

In a longitudinal general population sample with observed maternal behaviors at age 29 weeks, assessment of attachment security in the Strange Situation at age 14 months, and maternal reports of CU traits at age 2.5, 3.5, and 5.0 years, we showed that increased positive parenting reflecting both maternal sensitivity to distress and maternal positive regard in infancy were associated with reduced CU traits in early childhood. Sensitivity to distress and positive regard clearly had stronger effects than either sensitivity to nondistress or intrusiveness, and they acted synergistically so that the risk for high CU traits arose from the combination of low sensitivity to distress and low positive regard. Although maternal sensitivity to distress predicted attachment insecurity and disorganization, neither was associated with subsequent CU traits, thus providing no evidence for mediation of the effect by attachment status at 14 months. There was also no evidence that attachment status moderated the association between parenting and child CU traits. This is the first study to provide support for a specific role for two facets of positive parenting during infancy, sensitivity to distress and positive regard, in relation to CU traits over the preschool period, and to show that attachment security is not implicated in these early processes.

The findings are consistent with work with older children suggesting that parental responsiveness to distress may play a role in child empathy development (Davidov & Grusec, 2006; Humphreys et al., 2015). The findings are also consistent with the broader literature documenting associations between positive aspects of parenting and CU traits from the preschool period through to late childhood (e.g. Hawes et al., 2011; Waller et al., 2014). However, contrary to our predictions, sensitivity to distress was not unequivocally the strongest predictor of CU traits, and on balance maternal positive regard, irrespective of the infant's emotional state, may have been the stronger predictor. The finding of a significant interaction between sensitivity to distress and positive regard suggested that the risk for CU traits arises from a combination of lack of contingent responding to distress and lack of warmth. This needs replication, but the implication for intervention studies is that improvements in either parenting characteristic would be associated with lower CU traits.

In line with previous findings, lower maternal sensitivity to distress was significantly modestly associated with insecure attachment, with sensitivity to nondistress a nonsignificant predictor (Leerkes, 2011; McElwain & Booth‐LaForce, 2006). The same pattern of findings was true for disorganized attachment status. Neither insecure nor disorganized attachment at 14 months predicted later CU traits. In spite of the many differences in samples and measures between this study and the study of Pasalich et al. (2012), the contrast in findings may indicate that attachment status contributes to CU traits specifically in the context of conduct problems, or environmental risks associated with conduct problems. Alternatively, consistent with findings reported by Wagner et al. (2015) and Pasalich et al., attachment processes may contribute to risk of CU traits only after infancy. To maintain comparability with most other studies of attachment and externalizing problems we did not examine associations with dimensional indices of attachment. Given the evidence that attachment categories are not natural taxon's (Fraley & Spieker, 2003), a dimensional approach may have given a different result. It seemed therefore that the association between increased maternal sensitivity to distress and reduced CU traits was not mediated via attachment status, suggesting that there may be at least two pathways from maternal sensitivity to later developmental outcomes. One, mediated via attachment security may be specific to emotion regulation with a caregiver, while the other may entail the promotion of emotional and social understanding and responsiveness more generally.

The study was characterized by a number of strengths in the study design, sample and measurement. This was a prospective study of a consecutive sample from an antenatal clinic serving a defined geographical area. Sequential measurement of maternal parenting characteristics, infant attachment status, and child CU traits made it possible to conduct mediation analyses. We used several indicators of parenting, examining both their joint effect as a factor, and their additional independent effects, and their interaction. Parenting characteristics and infant attachment ratings were based on observations, and independent ratings were made blind to each other and to other measures. The problem of selection of sensitivity to distress measures by infant distress was addressed using a latent variable approach with the pattern of relatedness observed in dyads with all four parenting variables present used to predict the missing data on sensitivity to distress. We followed an approach previously used to combat low internal consistency in CU traits measures (Dadds, Hawes, Frost, & Fraser, 2005) using exploratory and confirmatory factor analysis on a widely used measure, the APSD, and other relevant developmentally appropriate items from early childhood problem behavior measures. This created measures with acceptable psychometric properties. Finally, we took a latent variable approach to modelling the CU traits outcome by combining reports from three time points throughout early childhood. This increased the robustness of the results by reducing the influence of measurement error. It also allowed us to examine a CU traits outcome that reflected persistence of CU traits, likely to be associated with poorer outcomes later in childhood.

Limitations of the study include that CU traits were assessed using mother‐report only. We sought to account for the effects of maternal mood on reporting, but could not rule out that mothers who are themselves less sensitive to distress may perceive their children as being less empathic. We also cannot exclude common genetic influences on maternal sensitivity and children's CU traits. The sample is almost exclusively White British so the findings may not be generalizable to other ethnic groups. The four parenting codes analyzed in this study were selected based on their prior use in publications from the NICHD sample, however, other parenting behaviors may be relevant, and for example, mothers who are low on sensitivity may in fact be exhibiting detachment. Both sensitivity to distress and to nondistress were coded from the same free‐play task and it is possible that distress occurring during a playful context may not be representative of maternal responses to distress in more threatening situations (Leerkes, 2011). A further possible limitation of the assessment of sensitivity to distress from a play procedure is that periods of distress are likely to occur over a minority of the overall observation period. This may lead to reduced validity where distress is brief. We sought to address this issue by comparing associations between sensitivity to distress and to nondistress, as an index of construct validity, across different durations of infant distress, and found they were very similar. Thus although we are not in a position to rule out an effect of duration of distress on the assessment of sensitivity, we did not find evidence for such an effect.

We have identified a possible specific mechanism involved in the early emergence of CU traits which may serve as a potential target for intervention, with the prospect that the relevant outcomes can be identified relatively soon after the intervention. We measured sensitivity at 29 weeks, however, maternal sensitivity measured even earlier has been linked to poorer child outcomes in other studies (e.g. 2 months; Hentges et al., 2011) which makes the case for examination of parent‐infant interaction and later outcomes with measurements at multiple points over the first year of life. Previous work has indicated that infants who show low eye gaze early in development may be an important target group for study and hence intervention, an important avenue for future work should focus on studying the interplay between maternal parenting characteristics and low eye gaze in samples of heightened risk across early development.

## Conclusion

In sum, this study provides further evidence that aspects of positive parenting are associated with reduced child CU traits. The findings are the first to indicate a specific role for maternal sensitivity to distress, and to show that attachment security or disorganization do not mediate this association. The findings have implications for research examining early developmental pathways to CU traits and for potential preventative intervention.

**Key points**

- Positive aspects of parenting such as parental warmth are known to be associated with lower CU traits, but evidence is lacking regarding the role of maternal sensitivity early in childhood.
- In a longitudinal general population study, mothers’ positive regard/warmth and sensitivity to infant distress at 7 months were associated with lower CU traits over the period 2.5 to 5.0 years. There was a significant interaction between the two, arising from the combination of low sensitivity to distress and low positive regard creating the risk for elevated CU traits.
- This is the first study to identify a link between the infants’ experiences of having had their emotions responded to empathically and lower CU traits. This link was not mediated by child attachment status.
- The findings provide a rationale for interventions to promote parental responsiveness to infant emotions and parental warmth to reduce later CU traits.

## Supporting information


**Appendix S1.** Creation of the age 3.5 years CU traits factor score.
**Table S1.** Standardized factor loadings from the CU and aggression CFA for ages 2.5 years, 3.5 years and 5 years.
**Table S2.** Age 3.5 years CFA models testing measurement invariance across sex.
**Table S3.** Comparison of the two‐factor CU traits and aggression model to a one‐factor model where all items load on the same factor.
**Table S4.** Summary statistics and spearmans correlations between the age 2.5, 3.5, and 5.0 years CU traits item mean scores.
**Table S5.** Pearsons correlations between the age 2.5, 3.5, and 5.0 years CU traits factor scores.

## Note

1 Spearman's rho correlations are used throughout because the sensitivity distributions were skewed.
