# Supplementary material for: Maternal sensitivity to distress, attachment and the development of callous‐unemotional traits in young children
Source: J Child Psychol Psychiatry. 2018 Jan 30;59(7):790–800. doi: 10.1111/jcpp.12867 (PMC6033174; doi:10.1111/jcpp.12867)
Supplement: Supplementary file 1 — Appendix S1. Creation of the age 3.5 years CU traits factor score. Table S1. Standardized factor loadings from the CU and aggression CFA for ages 2.5 years, 3.5 years and 5 years. Table S2. Age 3.5 years CFA models testing measurement invariance across sex. Table S3. Comparison of the two‐factor CU traits and aggression model to a one‐factor model where all items load on the same factor. Table S4. Summary statistics and spearmans correlations between the age 2.5, 3.5, and 5.0 years CU traits item mean scores. Table S5. Pearsons correlations between the age 2.5, 3.5, and 5.0 years CU traits factor scores. [file JCPP-59-790-s001.docx]

Online Supporting Information for: Maternal sensitivity to distress, attachment and the development of callous-unemotional traits in young children – by Wright et al.

Appendix S1: Creation of the age 3.5 years CU traits factor score

Rationale

Existing measures of CU traits were developed and validated with samples of children of mid to late childhood age. The majority of studies of children under 5 years of age have created hybrid measures of CU traits from other child problem behaviour measures. These scales have tended to comprise a small number of items and thus suffer from low internal consistency reliability. Dadds, Hawes, Frost, and Fraser (2005) supplemented the Antisocial Personality Screening Device (APSD; Frick & Hare, 2001) a widely used measure of CU traits in older samples, with the prosocial items from the Strengths and Difficulties Questionnaire (SDQ; Goodman, 1997) to improve the internal consistency of the measure. We have taken the same approach by supplementing the APSD with items from a number of child problem behaviour measures. We sought to establish a measure that is invariant across sex to allow the measure to be used to test for sex differences in associations between CU traits and other variables of interest.

Method

Following the approach used in Wright et al. (submitted) items from three different child problem behaviour measures were subjected to exploratory and confirmatory factor analyses. All six items from the CU subscale of the APSD were selected. Six items were selected from the Child Behaviour Checklist (CBCL; Achenbach & Rescorla, 2000) the item ‘doesn’t seem to feel guilty after misbehaving’ was not included due to similarity to the APSD item ‘feels bad or guilty when he/she does something wrong’. Finally, one item from the Brief Infant Toddler Socio-emotional Assessment (BITSEA; Briggs-Gowan, Carter, Irwin, Wachtel & Cicchetti, 2004) was included based on its similarity to the SDQ prosocial items. All items are rated on a three point scale (0, 1, 2). The same 13 items were considered for age 2.5 in Wright et al., 17 items were considered for age 5 as the SDQ prosocial items were available at that age. Also following on from the approach taken in Wright et al. we used 5 physical aggression items taken from the work of Baillargeon et al. (2007) to examine whether parents were reliably able to distinguish CU items from aggression items. All the items used are displayed in Table S1.

In the first stage of the analysis, the CU items were entered into an exploratory factor analysis for ordinal data (using the weighted least squares mean adjusted estimator [WLSM] and promax rotation) in Mplus version 7 (Muthen & Muthen, 2012). Items with a factor loading >.35 were retained. A series of multi-group two-factor CFA models were then used to examine for measurement invariance across sex in CU traits and aggression. The weighted least squares means and variance adjusted (WLSMV) estimator and Theta parameterization were used. In model 1 (configural model) the pattern of factor loadings were constrained to be the same for boys and girls, testing that the same items form the CU and aggression factors across sex. In model 2 (metric model) the individual factor loadings were constrained to be the same, testing whether the contribution of individual items varies by sex (weak factorial invariance). In model 3 (scaler model) the thresholds were also constrained to be the same, to examine whether the items perform the same across sex (strong factorial invariance). Full invariance is demonstrated when placing additional constraints on the model does not produce a significant worsening in model fit. The *DIFFTEST* command was used to evaluate whether a substantial change in model fit occurred as a result of imposing additional constraints, as well as the CFI change (ΔCFI). A non-significant chi-square difference test and a small CFI change (in which a decrease is no greater than .01) are considered indicative of invariance (Cheung & Rensvold, 2002). If a significant chi-square difference test is found, the modification indices are examined to determine which items failed the strong factorial variance assumption. In the absence of modification indices the individual items are checked for those showing the largest difference between boys and girls. The thresholds of these items are then allowed to vary freely and model fit is re-examined as a test of partial strong factorial invariance. Further, the modification indices are inspected for each model to check for cross-loading of CU items on the aggression factor. Items which fail the factorial invariance assumption or items with modification indices that indicate cross-loading will be evaluated for removal from the model.

CFA was then used to compare a two-factor CU and aggression model to a model where all CU and aggression items loaded on the same factor, to test whether parents could reliably distinguish CU traits items from aggression items. The two models were compared using the DIFFTEST command.

To avoid numerical problems associated with sparse data, where endorsement rates were < 1.5%, scores of 1 and 2 were collapsed to create binary variables. This was applied to the CU traits items ‘cruel to animals’, ‘shows little affection’ and unresponsive to affection’ and all of the aggression items. Although this generated adequate cell sizes in the sample as a whole, the analytic approach required adequate numbers in both males and females. Following the approach taken in Wright et al. ‘gets in many fights” was combined with the similar item “physically attacks others”, and “bites other children” was combined with the next rarest item “kicks other children”.

Results

*Exploratory factor analysis on the age 3.5 CU items.* The 13 CU items were entered into an EFA. Eigenvalues for first three factors were 4.8, 1.5, and 1.2, and the scree plot supported a one factor solution. All items gave factor loadings >.35 and so all items were retained.

*Confirmatory factor analyses testing measurement invariance.* The 13 CU items and the three aggression items were then tested for measurement invariance across sex. Model 1, the configural model, showed acceptable fit (RMSEA = .06, CFI = .91). However, the modification indices indicated that items ‘CBCL: cruel to animals’ should cross-load on the aggression factor for girls and ‘CBCL: selfish’ should cross-load on aggression for boys. These items were removed and a further configural model (Model 1b) was tested on the remaining 11 items and showed improved fit (RMSEA = .05, CFI = .96) with no further modification indices. The introduction of factor loading invariance with model 2 resulted in a very slight improvement in fit (RMSEA = .04, CFI = .96). However, the chi-square difference test was significant (*p* = .034) and inspection of the modification indices suggested that item ‘APSD: does not show feelings or emotions’ should load positively on the CU factor for boys and negatively for girls. In our previous analyses on the 5 year data this item did not show a factor loading >.35 in the initial EFA, and other studies have similarly not found this item to load sufficiently with the other APSD items (Dadds et al., 2005). Therefore we ran a third configural model (Model 1c) with this item removed. This model showed good, but not improved fit (RMSEA = .05, CFI = .96) and modification indices now indicated that item ‘BITSEA: tries to help others’ should cross-load on aggression. This item was removed, and a further configural model (Model 1d) was tested on the remaining 9 items. This model showed very good fit to the data (RMSEA = .01, CFI = 1.00) and presented no further modification indices. Factor loading invariance was then introduced with Model 2b, this model showed good fit (RMSEA = .02, CFI = 1.00) and a non-significant chi-square difference test indicated that imposing factor loading invariance did not significantly worsen the fit of the model (p = .245). The introduction of threshold invariance in Model 3 resulted in a slight improvement in fit (RMSEA = .01, CFI = 1.00) and the chi-square difference tests’ were non-significant for the comparison to Model 1d (*p* = .345) and Model 2b (*p* = .901). Therefore strong factorial invariance across sex was achieved on the remaining 9 items. The full model fit and comparison results are presented in Table S2.

*One- versus two-factor CFA models.* We then examined whether mothers’ could differentiate CU traits and aggression by comparing a one-factor CFA model where all the CU and aggression items loaded on one factor, to the two factor model, using the chi-square DIFFTEST. The model fit statistics and model comparison results are displayed in Table S2. The two-factor model showed the best fit (RMSEA = .01, CFI = 1.00) and the chi-square difference tests indicated that the two-factor model showed significantly better fit (p<.001). The standardised factor loadings are displayed in Table S1.

*Descriptive statistics for the age 2.5, 3.5 and 5 years CU traits measures.* Extracted factor scores were used for analysis but an item mean score for each age point was created to produce meaningful means and standard deviations, presented in Table S4. Table S4 shows the spearmans correlations between the item mean scores for the three age points and Table S5 shows the pearsons correlations between the factor scores.

References

Achenbach, T. M., & Rescorla, L. A. (2000) *Manual for the ASEBA preschool forms & profiles*. Burlington, VT: University of Vermont, Research Center for Children, Youth and Families.

Baillargeon, R. H., Zoccolillo, M., Keenan, K., Côté, S., Pérusse, D., Wu, H., et al. (2007). Gender differences in physical aggression: A prospective population-based survey of children before and after 2 years of age.*Developmental Psychology, 43*(1), 13-26. doi: 10.1037/0012-1649.43.1.13.

Briggs-Gowan, M., Carter, A., Irwin, J., Wachtel, K., & Cicchetti, D. (2004). The brief infant-toddler social and emotional assessment: Screening for social-emotional problems and delays in competence. *Journal of Pediatric Psychology, 29*(2), 143-155.

Cheung, G. W., & Rensvold, R. B. (2002). Evaluating goodness-of-fit indexes for testing measurement invariance. *Structural Equation Modeling, 9,* 233–255.

Dadds, M.R., Hawes, D.J., Frost, A., & Fraser, J. (2005). Disentangling the underlying dimensions of psychopathy and conduct problems in childhood: A community study. *Journal of Consulting and Clinical Psychology, 73*(3), 400-410.

Frick, P. J., & Hare, R. D. (2001). *The antisocial process screening device (APSD).* Toronto: Multi-Health Systems.

Goodman, R. (1997). The strengths and difficulties questionnaire: A research note. *Journal of Child Psychology and Psychiatry, 38*, 581-586.

Muthén, L. K., Muthén, B. O. (2012). *Mplus User’s Guide. Seventh Edition*. Los Angeles, CA: Muthén & Muthén

Wright, N., Sharp, H., Pickles, A., & Hill, J. (2017). Wright, N., Sharp, H., Pickles, A., & Hill, J. Measurement of callous-unemotional traits in very young children: a psychometric and validity study from 2.5 to 5.0 years. Manuscript submitted for publication.

**Table S1.** Standardised factor loadings from the CU and aggression CFA for ages 2.5 years, 3.5 years and 5 years

| **Items** | **Age 2.5** | **Age 3.5** | **Age 5** |
| --- | --- | --- | --- |
| **CU traits items** | | | |
| APSD 1: Concerned about the feelings of others (R) | .48 | .42 | .41 |
| APSD 2: Seems motivated to do his/her best in structured activities (R) | .61 | .37 |  |
| APSD 3: Is good at keeping promises (R) | .54 | .51 | .49 |
| APSD 4: Feels bad or guilty when he/she does something wrong (R) | .48 | .46 | .61 |
| APSD 5: Keeps the same friends (R) | .36 | .16 | .49 |
| APSD 6: Does not show emotions |  |  |  |
| CBCL 14. Cruel to animals | .93 |  | .59 |
| CBCL 58: Punishment doesn’t change his/her behavior | .62 | .74 | .68 |
| CBCL 67: Seems unresponsive to affection | .77 | .69 | .81 |
| CBCL 69: Selfish or won’t share | .42 |  |  |
| CBCL 70: Shows little affection toward people | .48 | .75 | .82 |
| CBCL 72: Shows too little fear of getting hurt |  | .49 |  |
| BITSEA 22. Tries to help if someone is hurt (R) | .69 |  |  |
| SDQ 1: Considerate of other people’s feelings (R) |  |  | .82 |
| SDQ 4: Shares readily with other children (R) |  |  | .60 |
| SDQ 9: Helpful if someone is hurt, upset or feelings ill (R) |  |  | .75 |
| SDQ 17: Kind to younger children (R) |  |  | .70 |
| SDQ 20: Often volunteers to help others (R) |  |  | .56 |
| **Aggression items** | | | |
| Hits other children | .76 | .89 | .94 |
| Bites other children/Kicks other children | .75 | .84 | .96 |
| Gets in many fights/Physically attacks others | .87 | .88 | .87 |

Note: *APSD = Antisocial Personality Screening Device, BITSEA = Brief Infant Toddler Social and Emotional Assessment, SDQ = Strengths and Difficulties Questionnaire*

**Table S2:** Age 3.5 years CFA models testing measurement invariance across sex

|  | **Parameters** | **Chi2(df)** | **p** | **RMSEA** | **RMSEA 90% C. I** | **CFI** |
| --- | --- | --- | --- | --- | --- | --- |
| Model 1a: configural | 147 | 386.05(147) | .001 | .06 | .06 - .07 | .91 |
| Model 1b: configural (modified) | 119 | 204.74(111) | .001 | .05 | .04 - .06 | .96 |
| Model 1c: Configural (modified) | 103 | 179.29(97) | .001 | .05 | .04 - .06 | .96 |
| Model 1d: Configural (modified) | 97 | 80.45(75) | .313 | .01 | .01 - .03 | 1.00 |
| Model 2a: metric | 105 | 217.67(125) | .001 | .04 | .03 - .05 | .96 |
| Metric 2b:  (modified) | 85 | 95.74(87) | .001 | .02 | .01 - .03 | 1.00 |
| Model 3: scalar | 70 | 108.93(102) | .301 | .01 | .01 - .03 | 1.00 |
| **Model 1b**  **vs Model 2a** |  | 25.11(14) | .034 |  |  |  |
| **Model 1d vs Model 2b** |  | 14.95(12) | .245 |  |  |  |
| **Model 1d vs Model 3** |  | 29.33(27) | .345 |  |  |  |
| **Model 2b vs Model 3b** |  | 8.54(15) | .901 |  |  |  |

**Table S3:** Comparison of the two-factor CU traits and aggression model to a one-factor model where all items load on the same factor

|  | **χ2 (df)** | **CFI** | **RMSEA** | **RMSEA C I** | **Chi2 diff test** |
| --- | --- | --- | --- | --- | --- |
| **Age 3.5 years** | | | | | |
| 1 factor | 116.75 (57)*** | .96 | .05 | .04 - .06 |  |
| 2 factor CU traits and aggression | 42.52(56)* | .1.00 | .01 | .00 - .03 |  |
|  |  |  |  |  | 30.183(1)*** |

****p < .001, *p < .05*

**Table S4:** Summary statistics and spearmans correlations between the age 2.5, 3.5, and 5.0 years CU traits item mean scores

|  | Age 2.5 CU traits | Age 3.5 CU traits | Age 5.0 CU traits |
| --- | --- | --- | --- |
| Age 2.5 CU item mean score | 1.00 |  |  |
| Age 3.5 CU traits item mean score | .51*** | 1.00 |  |
| Age 5 CU traits item mean score | .44*** | .54*** | 1.00 |
| Mean | .41 | .44 | .30 |
| SD | .25 | .27 | .24 |

Note. ****p <.001*

**Table S5:** Pearsons correlations between the age 2.5, 3.5, and 5.0 years CU traits factor scores

|  | Age 2.5 CU traits | Age 3.5 CU traits | Age 5.0 CU traits |
| --- | --- | --- | --- |
| Age 2.5 CU factor score | 1.00 |  |  |
| Age 3.5 CU traits factor score | .58*** | 1.00 |  |
| Age 5 CU traits factor score | .50*** | .59*** | 1.00 |

Note. ****p <.001*
